# Considering Preference‐Based Patient Participation—Scientific and Clinical Outcome Measures With the Patient Preferences for Patient Participation Tool

**DOI:** 10.1111/jep.70423

**Published:** 2026-03-26

**Authors:** Ann Catrine Eldh, Marcus Bendtsen

**Affiliations:** ^1^ Department of Health, Medicine and Caring Sciences Linköping University Linköping Sweden; ^2^ Department of Public Health and Caring Sciences Uppsala University Uppsala Sweden

**Keywords:** Bayesian statistics, patient participation, person‐centred care, preference‐based, statistical analysis

## Abstract

**Rationale:**

Integrated and person‐centred care is defined by several elements, one of them being the terms for patient involvement with respect to their needs and resources. We call this ‘patient participation’, emphasising the sharing component of mutual information and knowledge exchange and the shared actions of the patient and their healthcare professionals in making plans and decisions, framing goals, and providing for self‐care. Comprehensively addressing all aspects, including both patients' preferences for and experiences of participation, deserve support how to investigate and represent preference‐based patient participation.

**Objective:**

To offer theoretical and analytical guidance for the Patient Preferences for Patient Participation tool (the 4Ps), with procedures suitable for research and clinical evaluation.

**Method:**

The paper illustrates the planning and execution of analyses to illuminate preference‐based patient participation (or partial such participation, or lack thereof). Also, we discuss why, when, and how to address preferences for patient participation along with preference‐based measures. Two sets of reports are presented for research and clinical purposes.

**Results:**

By means of three cases across different healthcare contexts, we illustrate what these findings represent, and how to differentiate between insufficient, fair, and sufficient terms for preference‐based patient participation. Furthermore, the more detailed scale for such patient participation is considered in relation to the outcome measures of interventions in research and clinical improvement.

**Conclusion:**

The 4Ps is a means for evaluating preference‐based patient participation, though the matching of ordinal measures for preferences and experiences requires a careful and well‐informed approach to planning and executing data collection and statistical analyses. As an outcome measure of interventions to facilitate more person‐centred healthcare, researchers and clinicians should recognise theoretical and conceptual conditions. This enhances a recognition of patients' voice and choice; patients and staff can agree on how best to make use of resources and needs, improving health and healthcare engagement.

## 1 Introduction

Patient participation is anticipated in everyday healthcare [1, 2], although its odds in practice have been varied and unpredictable [3, 4, 5]. The conceptual core of ‘sharing’ [6, 7] suggests ‘participation’ emphasises the processes and outcomes through which a person engages in their health and healthcare [8, 9]. Despite a rising recognition of the need for a more person‐centred patient participation [10], lessened prospects for such participation are noted [11, 12]. To facilitate healthcare services that are both evidence‐based and person‐centred, a recognition of patients' resources and needs (i.e., preferences) alongside their experiences is required [13].

Patient participation is sometimes proposed to primarily represent being involved in healthcare related plans and decisions. A supplementary, marked conceptualisation is offered by what patient participation means to patients [8]. Person‐centred patient participation signifies both general *sharing of* and *sharing in* components, that is, the sharing of information, experience, and knowledge, and a sharing in activities for the benefit of the individual's health and healthcare [14]. In more detail, patient participation comprise:

- Verbalising one's experiences, such as the perception and understanding of one's symptoms, and/or of treatments; and
- being active in planning and/or executing health related activities, including setting goals and agreeing on procedures, performance of approved treatment, but also learning about and executing everyday selfcare behaviours.

While patient participation can also incorporate sharing in healthcare development and improvement processes [15], this paper focuses on the individual's engagement in their own health and healthcare.

Preference‐based patient participation is submitted as a response to the need to define person‐centred conditions for patient‐persons' involvement in their health and healthcare [16]. Paying attention to in what ways and to what extent an individual prefers to participate aids an understanding of their needs and resources (embodying capacity, capability, and priorities). A corresponding catch of experiences of participation enables the evaluation of whether efforts to facilitate participation were in alignment with the preferences.

Consequently, research and clinical measures for patient participation should signify preferences and experiences, while employing a conceptually sound idea. The more expanded evidence on patients' conceptualisation has facilitated the advancement of such a tool: the Patient Preferences for Patient Participation, in short, the 4Ps. The 4Ps was developed in response to the need of means matching ethical, semantic, conceptual, and legislative standards, as well as the lived experience of patients [17]. Following a series of qualitative studies with people with short‐ and long term conditions and healthcare interactions, 12 attributes of patient participation were described and addressed for representativeness and validity [17, 18, 19], as presented in Table 1.

**Table 1:** Attributes of the 4Ps and corresponding elements.

Order of attributes	Attribute of patient participation (represented as items of the 4Ps tool)	Overarching element	Construct
1	Being listened to (by the healthcare staff)	Imparting information	‘Sharing of’ (intellectual exchange of significance for one's health and healthcare)
2	My experiences being recognised		
3	Having reciprocal communication		
4	Telling about my symptoms/issues	Exchanging knowledge	
5	Having explanations as to my symptoms/issues		
6	Having explanations as to what will be/is done for me		
7	Learning what is planned for me	Arranging for healthcare	‘Sharing in’ (activities regarding one's health and healthcare)
8	Taking part in planning		
9	Phrasing my own (health) goals		
10	Knowing how to manage symptoms/issues	Accomplishing health and healthcare	
11	Managing (prescribed) treatments		
12	Performing self‐care		

In the 4Ps tool, the 12 attributes are presented as discrete items for patients to consider with respect to their preferences for and experiences of participation, respectively [14, 17, 18]. Completed by the patient person him‐ or herself, the tool can serve the individual and thus their healthcare professionals and team: details on whether the provision of conditions aligns with patients' preferences (or not) provide for a shared idea of the terms and extent of person‐centred (i.e., preference‐based) patient participation. The preference‐based patient participation score is manufactured by an appraisal of the patient's report of their preferences for and experiences of patient participation for each item of the 4Ps [14].

Consequently, the 4Ps tool is offered for evaluating healthcare services as well as research interventions and/or clinical improvement initiatives [16], with the principles for application further outlined in this paper.

Preferences for participation represent what one as a patient anticipates and needs; thus, preferences are to be recounted prospectively. Conversely, experiences are reported in hindsight, representing the provisions available for one's participation. While experiences are linked to the person, the delivery of conditions (for patients' participation) lies with the healthcare professionals and organisations (and is framed as such in the 4Ps). To enable completion of the 4Ps (and further investigations of preference‐based patient participation), the tool comes with four fixed response alternatives for each of its two sections (i.e., preferences for, and experiences of, participation, respectively). These are considered parallel in terms of their estimate:

- In the Preferences' section, the items are phrased in current tense, and each item is reported by means of one of the following alternatives: unimportant, somewhat important, very important, or crucial [for me to experience participation as a patient]. (The response alternative ‘crucial’ in the Preferences section was inserted to omit a prior ceiling effect of the 4Ps [18], and has been shown to eliminate such problems.)
- The Experiences section is reported in past tense, and is a response to whether the patient has experienced each attribute (i.e., item) ‘not at all’, ‘to some extent’, ‘to a large extent’, or ‘entirely’.

The analysis of both preferences and experiences requires a careful selection of principles, as does the representation of results. So far, valid versions are available in Swedish, English, and Norwegian; with the increasing use of the 4Ps across countries and settings, there is a call from scientists and clinicians for procedures to produce and convey findings and outcomes. Furthermore, any everyday healthcare dialogue with patients, as individuals or groups, also necessitates structures for translating statistical findings on preference‐based patient participation into lay language. With various statistical methods at hand, guidance on how to grasp and illustrate preference‐based patient participation is essential.

### 1.1 Aim

The aim of this paper is to provide theoretical and analytical guidance for the Patient Preferences for Patient Participation tool (the 4Ps), with procedures suitable for research and clinical purposes.

## 2 Methods

This section consists of two segments, corresponding to: (a) planning for the use of the 4Ps; and (b) the operationalisation, analysis, and representation of the 4Ps.

### 2.1 Planning

The 4Ps is a single‐item tool (not producing a sum‐score), requiring a recognition of match or gap between preference and experience per item. Rather than claiming that the higher the experience of patient participation, the better, the 4Ps serves person‐centredness, signifying whether services provided were in accord with the needs and resources of the person who is a patient. From a professional perspective, this helps staff understand whether they are spending time and effort on actions that are not benefiting a patient at a given time or within a certain process. A lack of priority regarding preferences also gambles with the patient's will and drive to participate, intellectually and/or matter‐of‐factly.

The 4Ps is protected by copyright to guarantee correct employment. It is available for free, following contact and sanctioned agreement with the first author. This includes a recognition that each item of the 4 P tool is to be considered separately and that the tool targets patient participation only (although in a comprehensive and exhaustive way). Furthermore, whether it is suggested for research, clinical evaluation, or quality improvement purposes, planning incorporates certain decisions regarding how the 4Ps tool is to be operationalised, which also dictates which statistical approaches are appropriate. In what follows, we will describe different operationalisations of the 4Ps tool and suggested statistical approaches, with examples from prior research.

When used for clinical purposes, the 4Ps items are all considered, with no discrepancy. However, for analytical purposes in research studies and improvement efforts where the 4Ps is to be used as an outcome measure, researchers should define which items are primary and secondary, respectively. This decision should be based on the contemporary study's aim and the theoretical core of the included intervention(s). This is to indicate which particular attributes of patient participation the intervention addresses—even if this does not indicate items as more or less important. By emphasising which 4Ps items are the main focus of a study, results can be presented and interpreted more clearly, and the risk of spurious findings is reduced.

An important aspect of this procedure is to prevent a rejection of an intervention in case it affects only a few (or even one) of the 12 items; it suffices a transparency in that the intervention may have been designed or convened to do exactly that. Even if an intervention can be designed which will be successful in improving all 12 attributes measured by the 4Ps, we have found that a clarification of what constructs and elements that guide the intervention and outcome measures favours the interpretability of analyses. Thus, the planning of a research study includes demarcating which 4Ps item(s) the study highlights and which 4Ps items the intervention is primarily designed to affect.

A note here: while setting up the data recording of the patient reports (for the selected statistical analysis program), certain attention should be given to the response alternatives for sections I and II, respectively, recognising that these are discrete. As such, even if converted to numerical values when registering the data prior to analysis, the preferences are ordered. Thus, ‘unimportant’, ‘somewhat important’, ‘very important’, or ‘crucial’ [for the respondents participation] may be listed as 1 through 4 in a statistical program, although this does not mean that they are converted to a higher data level. Likewise, experiences represented as ‘no [conditions for participation] at all’, or that conditions for participation occurred ‘to some extent’, ‘to a large extent’, or ‘entirely’) are ordered.

### 2.2 Operationalisation, Analysis, and Representation

The outcome measure constructed by a patient's response in terms of their preference for and experience of any of the 12 items of the 4Ps constitutes the degree of preference‐based patient participation for that specific attribute. With the response alternatives positioned in correspondence to each other between the sections for preferences and experiences, there are three global outcomes [14]:

- A *match* (i.e., preference‐based patient participation) between the alternative selected for preference and the alternative selected to represent experience. This includes correspondence between ‘unimportant’ and ‘not at all’, ‘somewhat important’ and ‘to some extent’, ‘very important’ and ‘to a large extent’, and ‘crucial’ and ‘entirely’.
- A mismatch representing *less* participation than preferred.
- A mismatch representing *more* participation than preferred.

The larger the gap between the response alternative selected for preference and the one for experience, the lesser the degree of preference‐based patient participation. A preference for being engaged, surpassing the individual's experience, indicates that the person's needs and resources were unnoticed or ignored. In contrast, an experience exceeding the preference may signify that staff have emphasised a certain kind and/or level of engagement, void of the needs and resources of the patient (or that there has been no communication at all, leaving the staff guessing as to the patient's preferences for participation).

**Figure 1:**
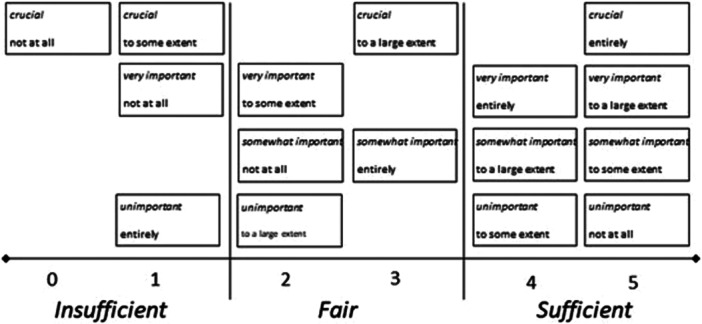
Ranks and levels of preference‐based patient participation. Overview of the 16 combinations for match or mismatch with the 4Ps, considering that the closer the match, the better, but more conditions for participation than preferred is better than less [14].

To help identify whether interventions (for clinical or research purposes) serve authentic improvement, the degree of preference‐based patient participation can be framed at one of two levels: as a six‐graded, numerical scale; or a three‐level, conceptual scale [14], as illustrated in Figure 1 and described in more detail below. While the statistical analyses we suggest intend to illustrate whether the patients' experiences match with their preferences, they also serve what any mismatch represents.

#### 2.2.1 The Six‐Graded Scale of Preference‐Based Participation

The six‐graded scale serves a detailed analysis of the 4Ps' capacity to illustrate the degree of preference‐based patient participation [14]. The ranks can be used, for example, to describe how person‐centred the provisions have been for a particular patient group or the effects of an intervention. When analysing outcomes on the six‐graded scale (0–5), one can either leverage the knowledge that the scale represents ordered categories and simply use ordinal regression, or one can use multinomial regression, and choose one score on the scale as the reference category (see the above paragraph on Planning), if the study was set up with such conditions.

When using ordinal regression, the output is more succinct, and the analysis can use the data more efficiently. However, if a perfect match is the only acceptable goal, then multinomial regression (using perfect match as reference category) will inform how close one is to this. Yet, if increased preference‐based participation is the goal, an ordinal model is a better fit; this should be the case when the 4Ps is used for clinical purposes, to measure quality or for improvement purposes.

***Case I***

Patients with severe chronic kidney failure are expected to engage in their health and healthcare in various ways. Furthermore, living with such a condition will require certain self‐care, such as compliance with fluid and food restrictions. Consequently, patient participation requires a sharing mode, with, for example, opportunities for learning as well as a commitment to learning.

In a quasi‐experimental study of a staff intervention designed to facilitate person‐centred participation in kidney care, the 4Ps was used as an outcome measure. Multilevel ordinal regression was used to estimate the effects of the intervention in terms of increased preference‐based participation. The analysis included outcomes from 245 patients across 9 sites and found no consistent evidence of effects of the intervention on the 4Ps. However, there was some evidence suggesting that the patients in the control group had slightly more matches between their preferences and experiences [20].

#### 2.2.2 The Three‐Grade Scale of Preference‐Based Participation

An alternative view of the six‐grade scale is formed by using a higher level of abstraction. This is offered primarily for clinical purposes, where three levels serve everyday dialogues on whether conditions provided for patient participation are fully‐, semi‐, or poorly person‐centred. This scale is ordinal in nature, based on empirical premises [14]:

- 0–1 on the six graded scale represents an insufficient level of preference‐based patient participation (calling for improved person‐centredness in terms of recognising patient preferences and providing conditions that can suffice for matching experiences).
- 2–3 illustrates the level of fair conditions for preference‐based patient participation. However, this is still indicative of a need to address mismatch in order to improve quality vis‐à‐vis person‐centredness.
- 4–5 indicates a level of sufficient conditions for preference‐based patient participation, with experiences aligning with patient preferences. This level is ideal, although it calls for sustainment (and ‘4’ still represents a slight need to elevate the conditions for being engaged in one's health and healthcare in accordance with one's preferences).

The three levels (as illustrated in Figure 1) can help clinical staff and managers appreciate that experiences that do not align with a patient's preferences can either represent that services did not provide what that person needed or was capable of in terms of their participation. Furthermore—it could be staff resources were at that point spent in vain. Both examples call for further attention and progress, preferably in a team approach with patient representation.

Statistical analysis choices for the three levels reflect those for the six‐grade scale. Again, it is commended to treat the outcome as ordinal, where the estimand is the odds of being higher up the scale. Still, an outcome can be treated as a categorical measure where the estimand represents the odds relative to a reference category—here undoubtedly the sufficient level.

#### 2.2.3 Match Scores

When studying the 4Ps tool match scores (operationalised by the comparison of a patient's preference vs. experiences), multinomial regression is recommended. Typically, we find it illustrative to use the perfect match category as reference category so that the odds ratios produced represent having less participation than preferred versus a perfect match, or more participation than preferred versus a perfect match.

***Case II***

Worldwide, thousands of people are faced with a need for hip surgery each year. While the surgical procedure is common, recovery and rehabilitation can vary from swift to complex. Notwithstanding, there are certain aspects that call for patient participation in the orthopaedic context, such as taking part of information on risks and self‐care, supposedly provided in consideration of the patient's state, including their health literacy and knowledge needs.

A trial including 17 Swedish orthopaedic sites serve as an example; the intervention sites were supported to facilitate implementation of evidence‐based guidelines for pre‐ and postoperative bladder care, which imply (presumably) person‐centred dialogues on toileting, mobilisation, pain, and pain management, etc [21]. Thus, the project hypothesised that an adoption of the guidelines would render staff more often involving patients in their own care.

Here, the 4Ps served as a secondary outcome measure to illustrate to what extent patient preferences for and experiences of participation matched. The match scores were compared between patients cared for in intervention and control sites, using multilevel multinomial regression with a perfect match as reference category.

Preliminary findings indicate that patients in intervention units had a lower odds of experiencing less than preferred (rather than preferred match) with respect to the first 4 P item (being listened to) both immediately after the intervention and 12 months later.

#### 2.2.4 Preferences for and Experiences of Patient Participation Scores, Respectively

Although preference‐based patient participation is the main objective studied with the 4Ps, separate supplementary analyses of the two sections are optional. Typically, this will supplement what signifies preferences for and experiences of patient participation, respectively. This is of interest in repeated measures, where such further command illuminates, for example, whether improved preference‐based patient participation was due to patients' experiences coming to better match their preferences, or if the patient preferences had changed.

An additional investigation of preferences helps in identifying whether and how they differ given certain patient group characteristics, on the subject of, for example, the progression of symptoms or treatment, or demographics like age or educational levels. Thus, while the correspondence between preferences and experiences aids research or clinical enterprises by mapping to what extent the context is person‐centred or not, the additional constitution of match versus mismatch aids in identifying which features must be primarily attended to.

For example, a regression analysis of demographic factors potentially associated with certain preferences can aid a better understanding of populations. To some extent, this is also the case for experiences of patient participation, although the map of provisos is not of general interest in and of itself, but remains pointless unless related to the person's preferences. Yet, deciphering a noted increase in person‐centredness in participation (following clinical and/or research interventions) requires further comparisons of experiences and preferences, respectively, over time. This will illuminate what has altered, aiding in the evaluation of the intervention. Still, preferences for patient participation can also be of general interest, helping healthcare professionals and organisations better understand the expectations of certain populations; this can, for example, be used to initiate a dialogue on participation when patients are unfamiliar with voicing their needs for engaging in health and/or healthcare. The response alternatives for preferences are discrete, and can be used to communicate whether one selects one or more particular items to indicate what is more or less vital for participation. Again, reports on patient preferences (and experiences) represent ordered categorical variables, and a good starting point for the statistical analysis of preferences is to model responses using ordinal regression.

If a better match between preferences for and experiences of patient participation following an intervention (for research or quality improvement purposes) has been identified, the further analyses of repeated measures of preferences and experiences, respectively, will indicate whether this is because the patients' experiences now align better with their preferences—or, if the patients' preferences have altered. The latter can indicate (anticipated) fluctuations in peoples' preferences for their participation in health and healthcare. For example, when living with a long‐term condition there are phases where one primarily prefers to partake in terms of learning and sharing experiences, and others in which a more active sharing in activities such as plans and engaging in prescribed treatments is preferred and manageable [22, 23]. Yet, altered preferences may indicate that the person who is a patient has come to comply with what staff promotes in terms of participation. Such a case merits further investigation, often with qualitative methodology, to better understand the depth and width of patients' experiences.

***Case III***

The PERHIT project [24] trialled an interactive web‐based self‐management system for patients with hypertension in Swedish primary care. The 4Ps was used as a secondary outcome, proposing that more opportunities for patients to learn about their condition, treatment, and self‐care, along with access to support of primary care staff, would enhance person‐centred engagement.

To illustrate differences in preferences among patients, responses to the first part of 4Ps were analysed using ordinal regression. Overall, we found that women had higher odds of preferring higher levels of participation than men (although women were, at the same time, less likely to experience preference‐based patient participation) [25].

#### 2.2.5 A Word on Regression Models and Estimation

Regression is a powerful statistical tool that allows us to estimate associations among quantifiable variables. It is straightforward to add adjustment variables to a regression model as required, as well as potential moderators or levels when data are clustered. When randomisation is part of the study design, regression models can be used to estimate causal relationships. *Ordinal* regression leverages the knowledge that responses are ordered to produce a single odds ratio, which indicates how the odds of responding higher on the scale differ given studied characteristics. In our studies, we have found that the proportional odds assumption underlying this approach is reasonable for responses to the 4Ps. Multinomial regression offers a higher degree of granularity, but at the cost of efficient data use and succinct results. These latter two features should not be undervalued when making decisions as for 4Ps analyses.

Our approach has mainly been to estimate effects and associations using Bayesian inference [26]. In this paradigm, parameters of regression models are assumed to have a distribution rather than a ‘true’ fixed value that we aim to estimate. While this may seem a subtle difference, it has major implications for the analytical approach and how results should be interpreted. In the Bayesian paradigm, we do not aim to reject or accept a null hypothesis—instead, we aim to estimate the distribution of the parameters. Put more plainly, we estimate a distribution of estimates and inspect the distribution (rather than focusing on a single effect or association estimate, and whether the null hypothesis can be rejected, or not). This allows for a more nuanced investigation of relationships, as we can answer the very straightforward question: What is the probability that the intervention had an effect? Such questions are impossible when taking a null‐hypothesis approach (with effects assumed to be fixed and known, and the data's plausibility used to reject the null or to fail to reject the null). The null‐hypothesis approach is neither easily understood nor congruent with what we as researchers are interested in knowing. Hence, our recommendation is to use Bayesian inference when analysing the 4Ps tool; it allows for the discovery of more subtle differences in responses and can lay important groundwork for future investigations [27].

## 3 Discussion

The 4Ps was designed by a rational method [28], and has thus evolved as a result of concept promotion—engaging people with first‐hand experience of the phenomenon from a patient perspective—over an extended period of time. While deemed valid [17, 18, 19], it suggests measures beyond prior tools by including both preferences for and experiences of patient participation, and also catch these aspects in a prospective and retrospective approach (rather than, e.g., posterior reports of patients' preferred engagement in clinical decisions [29]). As such, healthcare professionals and researchers can, for example, not only catch that women more often than men anticipated a higher degree of involvement in nursing care decisions [30], but address this in more depth in healthcare interactions and/or research interventions. In addition, while the 4Ps enables more than assessing in retrospect the individual's perceived reality and whether conditions for engagement were relevant [31, 32], it allocates the individual's anticipated participation, with opportunities for healthcare teams and organisations to consider their current practice. Thus, the tool enables appraisal of the person‐centredness of communication and procedures in healthcare, for now primarily at micro level, that is, patient and healthcare professional interactions [33]. This commands agreement as to when the patient should and can provide their preferences. A similar call is commended for when it is feasible for the patient to report on their contemporary experiences, with a fair chance of appropriate recall. All in all, access to a tool such as the 4Ps will suffice neither clinical improvements nor research procedures in and of itself, but improvements call for implementation efforts, considering context along with the tool, and for additional attention to facilitate favourable actions [34, 35].

Increasing demands for healthcare, due to demographic changes, including, for example, more people with complex and multiple conditions, and further access to more sophisticated knowledge and the means to detect and treat conditions, call for both evidence‐based and person‐centred performance of organisations and individuals. Though both evidence‐based practice and person‐centred care are important, only one or the other will not ensure optimal quality. Rather, the challenge rests with the ‘and’: joining evidence‐based procedures with attitudes and actions that emphasise the needs and resources of the person enacting the patient role. Hitherto, patient participation has been suggested to emphasise the latter, although it is often tapered to self‐determination in relation to healthcare; that is, the person's involvement in planning for and making decisions on healthcare [36, 37]. We suggest the 4Ps as an additional means, linking autonomy to how and to what extent one is able to engage in health and healthcare at a given time. As such, recognition of patient preferences and experiences informs an evidence‐based practice, incorporating the individual's capacities and needs [38].

Nevertheless, an autonomous recognition of how and to what degree one prefers to engage in health and healthcare may challenge current and future healthcare. The call for effective services unavoidably encourages people to engage in their health and healthcare at one point or another. With the 4Ps, the preferences (of a person who is patient) to participate can be set to ‘not at all’ for many, most, or even all 12 aspects of their engagement, stirring a potential conflict of self‐determination and ethics. Though such a report is rare, it can hypothetically occur. In such case, we suggest appreciating what may seem like submissive preferences for patient participation by considering the full range of attributes of the 4Ps—if one or more items are indicated as more important than “not at all”, address those. Furthermore, recognise that although an endpoint from a professional perspective may be a future sharing in activities, more important at the current point of a healthcare trajectory for a patient may be being listened to, learning of, and understanding. These are all attributes of patient participation, and a foundation for further actions, including managing treatment and selfcare.

Being open to what particular attributes of participation that are suggested as important or vital at a certain point of care by patients with the 4Ps will enable a learning opportunity for professionals, especially if collated with people having a similar condition/situation. An accumulated further understanding of preference‐based patient participation aids a professional discussion with patients: even though a particular attribute was noted as unimportant for their participation, the knowledgeable staff can point out potential consequences for lesser engagement, and initiate a dialogue on the budding transition, inquiring as to what support the person who is a patient needs to become more involved in essential perspectives [39, 40]. Such ventures can provide for learning opportunities and prevent ineffective procedures.

Thus, the person‐centred ambition of the 4Ps extends to address the challenges of patient non‐participation [3] and/or self‐determination [41]. The individual capable of voicing their preferences and experience as a patient will recognise the necessity of sharing of and sharing in aspects related to their health and healthcare—that is, executing patient participation. Still, their participation will likely differ depending on what condition the person has, what type of healthcare contact it is, and the purpose of the healthcare interaction. Consequently, we suggest preference‐based patient participation fluctuates, which calls for careful measures of what attributes are selected, and what has altered for an individual. The 4Ps sustains a recognition of needs and resources for participation at given points of time and processes [42]. Yet, the implementation of attitudes and routines to suffice preference‐based patient participation calls for efforts beyond the 4Ps, but helps identify what works, for whom, and when. Still, further inquiries are required to understand why (or why not) certain healthcare services are person‐centred [43], where the 4Ps tool can detect barriers and enablers significant to person‐centred patient participation. While attempts to alter attitudes, behaviours, and routines are complex, requiring resources to map, report, and evaluate processes and outcomes [44, 45], the 4Ps is a means for mapping and reporting, and thus of evaluating preference‐based patient participation, both in research and clinical enterprises. We propose this paper for use in analysing, representing, and reporting the 4Ps in such studies.

## 4 Conclusion

Enabling patients, staff, and healthcare organisations to gain a better comprehension of what is and what serves as person‐centred patient participation, the 4Ps offers both a clinical and research measure for preference‐based patient participation. The matching of ordinal measures for preferences for and experiences of participation requires careful and well‐informed approaches to planning and executing data collection and statistical analyses. Hence, depending on the aim and procedures of the study or evaluation, multilevel ordinal regression, multilevel multinomial regression, or ordinal regression should apply.

Furthermore, as an outcome measure of interventions to facilitate more person‐centred healthcare, researchers and clinicians ought to recognise the theoretical and conceptual conditions for the tool, together with the basics of the intervention it evaluates. Accurate conduct will provide the potential for a sustained recognition of patients' voice and choice, through which patients and staff can make better use of resources and needs, for the benefit of improved health and healthcare engagement.

## Author Contributions

A.C.E. and M.B. conceptualised and wrote the manuscript, agreeing on the final version.

## Funding

The authors received no specific funding for this work.

## Ethics Statement

The studies referred to in the cases were approved by the Swedish Ethical Review Authority.

## Consent

Not applicable; the manuscript reflects cases from prior studies, all in which study participants had provided informed consent.

## Conflicts of Interest

The 4Ps tool is protected by copyright and available free of charge following agreement with the first author.

## Data Availability

The data contributing to the original analyses are available as per the standards of each study report and journal.
